# On the topology of surfaces with the generalised simple lift property

**DOI:** 10.1007/s10711-019-00455-z

**Published:** 2019-05-06

**Authors:** Francesca Tripaldi

**Affiliations:** Department of Mathematics and Statistics, University of Jyväskylä, 40014 Jyvaskyla, Finland

**Keywords:** Minimal lamination, Minimal surfaces, Colding and minicozzi theory, Simple lift property, 53A10, 51H05

## Abstract

In this paper, we study the geometry of surfaces with the generalised simple lift property. This work generalises previous results by Bernstein and Tinaglia (J Differ Geom 102(1):1–23, 2016) and it is motivated by the fact that leaves of a minimal lamination obtained as a limit of a sequence of property embedded minimal disks satisfy the generalised simple lift property.

## Introduction

Motivated by the work of Colding and Minicozzi [3–6] and Hoffman and White [8] on minimal laminations obtained as limits of sequences of properly embedded minimal disks, in [1] Bernstein and Tinaglia introduce the concept of the simple lift property. Interest in these surfaces arises because leaves of a minimal lamination obtained as a limit of a sequence of properly embedded minimal disks satisfy the simple lift property. In [1] they prove that an embedded minimal surface $\Sigma \subset \Omega$ with the simple lift property must have genus zero, if $\Omega$ is an orientable three-manifold satisfying certain geometric conditions. In particular, one key condition is that $\Omega$ cannot contain closed minimal surfaces.

In this paper, we generalise this result by taking an arbitrary orientable three-manifold $\Omega$ and introducing the concept of the generalised simple lift property, which extends the simple lift property in [1]. Indeed, we prove that leaves of a minimal lamination obtained as a limit of a sequence of properly embedded minimal disks satisfy the generalised simple lift property and we are able to restrict the topology of an arbitrary surface $\Sigma \subset \Omega$ with the generalised simple lift property.

Among other things, we prove that the only possible compact leaves of a minimal lamination obtained as limits of a sequence of properly embedded minimal disks are the torus in the orientable case, the Klein bottle and the connected sum of three and four projective planes in the non-orientable case.

## Notation and definitions

Throughout the paper, we will assume $\Omega$ to be an open subset of an orientable three-dimensional Riemannian manifold (*M*, *g*). We denote by ${dist}^\Omega$ the distance function on $\Omega$ and by $\exp ^\Omega$ the exponential map. Therefore, we have

$$\begin{aligned} \exp ^\Omega _p:{\mathbb {B}}_r(0)\rightarrow {\mathcal {B}}_r(p)\,, \end{aligned}$$

where ${\mathbb {B}}_r(0)$ is the Euclidean ball in ${\mathbb {R}}^3$ of radius *r* centred at the origin, and ${\mathcal {B}}_r(p)$ is the geodesic ball in *M* of radius *r* centred at $p\in \Omega$.

For an embedded surface $\Sigma$, we write

$$\begin{aligned} \exp ^\perp :N\Sigma \rightarrow \Omega \end{aligned}$$

to denote the norma exponential map, where $N\Sigma$ is the normal bundle.

If $N\Sigma$ is trivial, then we say that $\Sigma$ is *two-sided*, otherwise we say that $\Sigma$ is *one-sided*. As $\Omega$ is oriented, $\Sigma$ being two-sided is equivalent to saying that $\Sigma$ is orientable.

Let us fix a subset $U\subset N\Sigma$, then we define

$$\begin{aligned} {\mathcal {N}}_U(\Sigma ):=\exp ^\perp (U)\,. \end{aligned}$$

The set ${\mathcal {N}}_U(\Sigma )$ is *regular* if there is an open set *V* with $U\subset V$ such that $\exp ^\perp :V\rightarrow {\mathcal {N}}_V(\Sigma )$ is a diffeomorphism. If ${\mathcal {N}}_U(\Sigma )$ is regular, then the map $\Pi _\Sigma :{\mathcal {N}}_U(\Sigma )\rightarrow \Sigma$, given by the nearest point projection, is smooth and for any $(q,\mathbf {v})\in T{\mathcal {N}}_U(\Sigma )$, there is a natural splitting

$$\begin{aligned} \mathbf {v}=\mathbf {v}^\perp +\mathbf {v}^T\,, \end{aligned}$$

where $\mathbf {v}^\perp$ is orthogonal to $\mathbf {v}^T$, and $\mathbf {v}^T$ is perpendicular to the fibres of $\Pi _\Sigma$.

We say that such $\mathbf {v}$ is $\delta$-*parallel* to $\Sigma$ if

$$\begin{aligned} \vert \mathbf {v}^\perp \vert \le \delta \vert \mathbf {v}\vert \;\text { and }\;\frac{1}{1+\delta }\vert \mathbf {v}^T\vert \le \vert d(\Pi _\Sigma )_q(\mathbf {v})\vert \le (1+\delta )\vert \mathbf {v}^T\vert \,. \end{aligned}$$

Given $\epsilon >0$, we set $U_\epsilon :=\lbrace (p,\mathbf {v})\in N\Sigma \mid \vert \mathbf {v}\vert <\epsilon \rbrace$ and define ${\mathcal {N}}_\epsilon (\Sigma )$, the $\epsilon$-neighbourhood of $\Sigma$, to be ${\mathcal {N}}_{U_\epsilon }(\Sigma )$. If $\Sigma$ is an embedded smooth surface and $\Sigma _0\subset \Sigma$ is a pre-compact subset, then $\exists \,\epsilon >0$ so that ${\mathcal {N}}_\epsilon (\Sigma _0)$ is regular.

Given a fixed embedded surface $\Sigma$ and $\delta \ge 0$, we say that another embedded smooth surface $\Gamma$ is a *smooth*$\delta$-*graph* over $\Sigma$ if there exists an $\epsilon >0$ such that:

1. ${\mathcal {N}}_\epsilon (\Sigma )$ is a regular $\epsilon$-neighbourhood of $\Sigma$;
2. either $\Gamma$ is a proper subset of ${\mathcal {N}}_\epsilon (\Sigma )$ or $\Gamma$ is a proper subset of ${\mathcal {N}}_\epsilon (\Sigma )\setminus \Sigma$;
3. for all $(q,\mathbf {v})\in T\Gamma$ is $\delta$-parallel to $\Sigma$.

We say that a smooth $\delta$-graph $\Gamma$ over $\Sigma$ is a *smooth*$\delta$-*cover* of $\Sigma$, if $\Gamma$ is connected and $\Pi _\Sigma (\Gamma )=\Sigma$.

Let $\gamma :[0,1]\rightarrow \Sigma$ be a smooth curve in $\Sigma$. We will also denote the image of such $\gamma$ as $\gamma$.

We say that a curve ${\widehat{\gamma }} :[0,1]\rightarrow {\mathcal {N}}_\delta (\gamma )$ is a $\delta$*-lift* of $\gamma$ if

- $${\mathcal {N}}_\delta (\gamma )$$ is regular;
- $$\Pi _\Sigma \circ {\widehat{\gamma }} =\gamma $$;
- for all $t\in [0,1]$, $({\widehat{\gamma }} (t),{\widehat{\gamma }} '(t))$ is $\delta$-parallel to $\Sigma$.

This definition extends to piece-wise $C^1$ curves in an obvious manner.

## The generalised simple lift property for a finite number of curves

Let us introduce the concept of lifts of curves into embedded disks.

### Definition 3.1

Generalised simple lift property.

Let $\Sigma$ be a surface in $\Omega$. Then $\Sigma$ has the *generalised simple lift property* if, for any $\delta >0$ and for any $p\in \Sigma$, the following holds.

Given $\gamma _1,\ldots ,\gamma _n:[0,1]\rightarrow \Sigma$ a collection of *n* arbitrary smooth curves, and any pre-compact open subset $U\subset \Sigma$ such that $\gamma _1\cup \cdots \cup \gamma _n\subset U$, there exist $t_i\in [0,1]$ for which $\gamma _i(t_i)=p$ for any $i=1,\ldots ,n$, as well as:

i. a constant $$\epsilon =\epsilon (U,\delta )>0$$;
ii. $\Delta \subset \Omega$ an embedded disk;
iii. $${\widehat{\gamma }} _i:[0,1]\rightarrow {\mathcal {N}}_\epsilon (U)$$$$\delta $$-lifts of $$\gamma _i$$

such that

1. $${\widehat{\gamma }} _i\subset \Delta \cap {\mathcal {N}}_\epsilon (U)$$;
2. $\Delta \cap {\mathcal {N}}_\epsilon (U)$ is a $\delta$-graph over *U*;
3. there exists a point $q\in {\mathcal {N}}_\epsilon (p)\cap \Delta$ such that $q\in {\widehat{\gamma }} _i$ for every $i=1,\ldots ,n$;
4. the connected component of $\Delta \cap {\mathcal {N}}_\epsilon (U)$ containing ${\widehat{\gamma }} _i$ is a $\delta$-cover of *U*.

The union ${\widehat{\gamma }} _1\cup \cdots \cup {\widehat{\gamma }} _n$ is called the *generalised simple*$\delta$*-lift of*$\gamma _1\cup \cdots \cup \gamma _n$*pointed at* (*p*, *q*) *into*$\Omega$.

One should notice that the embedded disk $\Delta \subset \Omega$ that the definition implies exists will depend on the choice of the constant $\delta >0$, the *n* curves $\gamma _1,\ldots ,\gamma _n$ and the pre-compact subset $U\subset \Sigma$ that contains the curves. Notation wise, throughout this paper, when studying a lift of *n* given curves $\gamma _1,\ldots ,\gamma _n$, if we want to highlight the dependence of the construction on the choice of curves, we will denote the embedded disk $\Delta$ that contains the generalised simple $\delta$-lift of $\gamma _1\cup \cdots \cup \gamma _n$ by $\Delta (\gamma _1,\ldots ,\gamma _n)$.

A surface with the generalised simple lift property is one for which, in an effective sense, the universal cover of the surface can be properly embedded as a disk near the surface. For this reason, to understand the topology of the surface $\Sigma$, it is important to understand the lifting behaviour of closed curves.

With this in mind, we give the following definition.

### Definition 3.2

Closed and open lift property.

Let $\Sigma \subset \Omega$ be an embedded surface with the generalised simple lift property. If $\gamma : [0,1]\rightarrow \Sigma$ is a smooth closed curve, then $\gamma$ has the *open lift property* if there exists a $\delta _0>0$ so that, for all $\delta _0>\delta >0$, $\gamma$ does not have a closed generalised simple $\delta$-lift ${\widehat{\gamma }} :[0,1]\rightarrow {\mathcal {N}}_\delta (\Sigma )$. Otherwise, $\gamma$ has the *closed lift property*.

If a closed curve $\gamma$ has the closed lift property, then there is a sequence $\delta _i\rightarrow 0$ so that there are closed simple $\delta _i$-lifts ${\widehat{\gamma }} _i$ of $\gamma$.

If it is possible to choose the lifts of a curve $\gamma$ to be embedded (and in particular non-intersecting), we say $\gamma$ has the *embedded (closed/open) lift property*.

### Remark 3.3

In Proposition 3.4 below and in Lemma 4.2 we will be constructing the lift of two (or more) simple closed curves intersecting at one point by considering the union of these curves as a single curve.

In order to fix the notation, let us assume we want to lift two curves $\alpha ,\,\beta :[0,1]\rightarrow \Sigma$ intersecting in one point $\lbrace p\rbrace =\alpha \cap \beta$. Then we will denote by $\mu :=\alpha \circ \beta$ the curve parametrised as $\mu :[0,1]\rightarrow \Sigma$ with

$$\begin{aligned} \mu \big \vert _{[0,t_0]}=\tilde{\alpha }\,,\;\mu \big \vert _{[t_0,1]}=\tilde{\beta } \end{aligned}$$

where $\tilde{\alpha }=\alpha (\frac{t}{t_0}):[0,t_0]\rightarrow \Sigma$ and $\tilde{\beta }=\beta (\frac{t-t_0}{1-t_0}):[t_0,1]\rightarrow \Sigma$ are appropriate reparametrisations of $\alpha$ and $\beta$ respectively, so that $\mu (0)=\mu (t_0)=\mu (1)$, for some $t_0\in (0,1)$.

The proposition below, which we will call *Lifting Lemma*, is analogous to Proposition 4.4 in Bernstein and Tinaglia’s paper [1].

### Proposition 3.4

**Lifting lemma**

Let $\Sigma \subset \Omega$ be an embedded surface with the generalised simple lift property. Let us take into consideration two closed, smooth curves

$$\begin{aligned} \alpha :[0,1]\rightarrow \Sigma \;\text { and }\;\beta :[0,1]\rightarrow \Sigma \end{aligned}$$

satisfying the following properties:

1. $$\alpha \cap \beta =\lbrace p\rbrace $$, where $$p=\alpha (0)=\beta (0)$$;
2. $\exists \,U\subset \Sigma$ a two-sided pre-compact open set that contains both curves, i.e. $\alpha \cup \beta \subset U$;
3. for this choice of $U\subset \Sigma$, there exists a $\delta >0$ for which the embedded disk $\Delta =\Delta (\alpha ,\beta )\subset \Omega$ given by Definition 3.1 contains open lifts for both $\alpha$ and $\beta$.

Then the curve $\mu :=\alpha \circ \beta \circ \alpha ^{-1}\circ \beta ^{-1}$ has a closed lift into this disk $\Delta (\alpha ,\beta )$.

If, in addition, both $\alpha$ and $\beta$ have embedded open lifts in $\Delta (\alpha ,\beta )$, then one of the following curves has an embedded closed lift in $\Delta (\alpha ,\beta )$:

$$\begin{aligned} \mu \;,\;\alpha \circ \beta \;,\;\beta \circ \alpha ^{-1}\,. \end{aligned}$$

### Proof

Let us take into consideration the curve $\mu =\alpha \circ \beta \circ \alpha ^{-1}\circ \beta ^{-1}$ as defined above.

Since $\Sigma$ has the generalised simple lift property, then for any $\delta >0$ there exist:

i. a positive constant $$\epsilon >0$$;
ii. an embedded disk $\Delta =\Delta (\alpha ,\beta )$;
iii. ${\widehat{\mu }} :[0,1]\rightarrow {\mathcal {N}}_\delta (U)$ a $\delta$-lift of $\mu$;

such that $\Delta (\alpha ,\beta )\cap {\mathcal {N}}_\epsilon (U)$ is a $\delta$-graph over *U*, ${\widehat{\mu }} \subset \Delta$, and $\Gamma$, the connected component of $\Delta (\alpha ,\beta )\cap {\mathcal {N}}_\epsilon (U)$ containing ${\widehat{\mu }}$, is a $\delta$-cover of *U*.

By assumption, we can consider the embedded disk $\Delta (\alpha ,\beta )$ which contains opens lifts of both $\alpha$ and $\beta$.

By re-parametrising appropriate restrictions of ${\widehat{\mu }}$, we can write ${\widehat{\mu }} ={\widehat{\alpha }} \circ {\widehat{\beta }} \circ {\widehat{\alpha ^{-1}}} \circ {\widehat{\beta ^{-1}}}$, where the ${\widehat{\alpha }} ,\,{\widehat{\beta }} ,\,{\widehat{\alpha ^{-1}}} ,\,{\widehat{\beta ^{-1}}} :[0,1]\rightarrow \Gamma$ are the $\delta$-lifts of $\alpha ,\,\beta ,\,\alpha ^{-1}$ and $\beta ^{-1}$ respectively.

Let us now pick a small simply-connected neighbourhood *V* of the point $p=\mu (0)$ such that $V\subset U$. By construction, $\Delta (\alpha ,\beta )$ is an embedded disk, which means that we can order by height the components of $\Pi _\Sigma ^{-1}(V)\cap \Delta (\alpha ,\beta )$, where $\Pi _\Sigma$ is the usual projection map onto $\Sigma$. We will denote these ordered components as $\Pi _\Sigma ^{-1}(V)\cap \Delta (\alpha ,\beta )=\lbrace {\widehat{V}} (1),\ldots ,{\widehat{V}} (n)\rbrace \,$. The number *n* of components will of course depend on the choice of $\delta >0$ and $\Delta (\alpha ,\beta )$.

By construction, we then have:

$$\begin{aligned} \Pi _\Sigma \big (\,{\widehat{\alpha (0)}} \,\big )&=\Pi _\Sigma \big (\,{\widehat{\alpha (1)}} \,\big )=\Pi _\Sigma \big (\,{\widehat{\beta (0)}} \,\big )=\Pi _\Sigma \big (\,{\widehat{\beta (1)}} \,\big )=\Pi _\Sigma \big (\,{\widehat{\alpha ^{-1}(0)}} \,\big )=\\ {}&=\Pi _\Sigma \big (\,{\widehat{\alpha ^{-1}(1)}} \,\big )=\Pi _\Sigma \big (\,{\widehat{\beta ^{-1}(0)}} \,\big )=\Pi _\Sigma \big (\,{\widehat{\beta ^{-1}(1)}} \,\big )=p\,. \end{aligned}$$

Without loss of generality, one can assume ${\widehat{\mu }}$ to be the generalised simple $\delta$-lift of $\mu$ pointed at (*p*, *q*) with $q={\widehat{\alpha (0)}}$. Moreover, a priori, these points will all belong to different components of $\Pi _\Sigma ^{-1}(V)\cap \Delta (\alpha ,\beta )$ and we will denote them as:

- $${\widehat{p}} (0):={\widehat{\alpha (0)}} =q\,$$;
- $${\widehat{p}} (1):={\widehat{\alpha (1)}} ={\widehat{\beta (0)}} \,$$;
- $${\widehat{p}} (2):={\widehat{\beta (1)}} ={\widehat{\alpha ^{-1}(0)}} \,$$;
- $${\widehat{p}} (3):={\widehat{\alpha ^{-1}(1)}} ={\widehat{\beta ^{-1}(0)}} \,$$;
- $${\widehat{p}} (4)={\widehat{\beta ^{-1}(1)}} \,$$;

so that ${\widehat{p}} (j)\in {\widehat{V}} (l)$, where *l* is a function of *j* over the natural numbers, that is $l=l(j)\in {\mathbb {N}}$.

Using this function *l*, we will study the signed number of sheets between the end points of the lifts of the curves $\alpha ,\,\alpha ^{-1},\,\beta$ and $\beta ^{-1}$:

$$\begin{aligned} m[\alpha ]:=l(1)-l(0);\\ m[\beta ]:=l(2)-l(1);\\ m[\alpha ^{-1}]:=l(3)-l(2);\\ m[\beta ^{-1}]:=l(4)-l(3)\,. \end{aligned}$$

By assumption, both ${\widehat{\alpha }}$ and ${\widehat{\beta }}$ are open lifts, so that $m[\alpha ],\,m[\beta ]\ne 0$, which also implies $m[\alpha ^{-1}],\,m[\beta ^{-1}]\ne 0$.

We will now prove that $m[\alpha ]=-m[\alpha ^{-1}]$ and $m[\beta ]=-m[\beta ^{-1}]$, and therefore that ${\widehat{\mu }}$ is closed.

Let us consider the two following cases separately:

- $$m[\alpha ]\cdot m[\beta ] >0$$

Without loss of generality, we can assume in this case that both numbers are positive: $m[\alpha ],m[\beta ] >0$. Then, using the fact that the disk $\Delta (\alpha ,\beta )$ is embedded and that *U* is two-sided, one can consider a disjoint family of *parallel lifts* of $\alpha$, which we will denote by ${\widehat{\alpha }} [i]$. The first member of this family is ${\widehat{\alpha }} [0]={\widehat{\alpha }}$ and the subsequent representatives of the family are those lifts ${\widehat{\alpha }} [i]$ of $\alpha$ such that ${\widehat{\alpha }} [i](0)$ will belong to ${\widehat{V}} \big (l(0)+i\big )$, which is the lift that starts *i* sheets above ${\widehat{\alpha (0)}} =q$. By the embeddedness of $\Delta (\alpha ,\beta )$ and the two-sidedness of *U*, the signed number of graphs between ${\widehat{\alpha }} [0](t)$ and ${\widehat{\alpha }} [i](t)$ is constant in *t*, so that also the lifts ${\widehat{\alpha }} [i]$ also have endpoints *i* sheets above the endpoint of ${\widehat{\alpha }}$.

Clearly, the lifts ${\widehat{\alpha }} [i]$ are well-defined as long as $i\le m[\beta ]$. Furthermore, ${\widehat{\alpha }} [m[\beta ]]$ has end point which is the same as the end point of ${\widehat{\beta }}$. Let us now take into consideration ${\widehat{\alpha }} [m[\beta ]]^{-1}$. This is a lift of $\alpha ^{-1}$ that starts at ${\widehat{\beta (1)}}$, which means that ${\widehat{\alpha }} [m[\beta ]]^{-1}$ and ${\widehat{\alpha ^{-1}}}$ must coincide. This then implies that $m[\alpha ]=-m[\alpha ^{-1}]$.

Repeating the same argument for ${\widehat{\beta }} [-m[\alpha ]]$ and ${\widehat{\beta }}$ shows that $m[\beta ]=-m[\beta ^{-1}]$.

- $$m[\alpha ]\cdot m[\beta ] <0$$

In this case, we can assume without loss of generality that $m[\alpha ]>0$ and $m[\beta ]<0$.

Let us first assume that $m[\alpha ]+m[\beta ]+m[\alpha ^{-1}]=:M\ge 0$, which means that the end point of ${\widehat{\alpha ^{-1}}}$ is not below the initial point of ${\widehat{\alpha }}$: it is *M* sheets above ${\widehat{\alpha }}$. Repeating the same argument as in the previous case, we can take into consideration the parallel lift of ${\widehat{\alpha ^{-1}}}$ whose endpoint is the initial point of ${\widehat{\alpha }}$, namely ${\widehat{\alpha ^{-1}}} [-M]$. The lift ${\widehat{\alpha ^{-1}}} [-M]^{-1}$ will then be a lift of $\alpha$ and it coincides with ${\widehat{\alpha }}$, which implies as before that $m[\alpha ]=-m[\alpha ^{-1}]$. Therefore the initial assumption $m[\alpha ]+m[\beta ]+m[\alpha ^{-1}]\ge 0$ leads to a contradiction, since by hypothesis $m[\beta ]<0$.

It is then the case that $m[\alpha ]+m[\beta ]+m[\alpha ^{-1}]=:M < 0$. Again, we can take into consideration the parallel lift of ${\widehat{\alpha }}$ whose start point coincides with the endpoint of ${\widehat{\alpha ^{-1}}}$, namely ${\widehat{\alpha }} [M]$. Therefore ${\widehat{\alpha }} [M]^{-1}$ is the lift of $\alpha ^{-1}$ with the same endpoint as ${\widehat{\alpha ^{-1}}}$: ${\widehat{\alpha }} [M]^{-1}$ and ${\widehat{\alpha ^{-1}}}$ coincide, and so $m[\alpha ]=-m[\alpha ^{-1}]$. The same argument shows that in this case $m[\beta ]=-m[\beta ^{-1}]$.

Finally, if $\alpha$ and $\beta$ have embedded open lifts in $\Delta (\alpha ,\beta )$, then, because they meet at only one point in $\Sigma$, the curves ${\widehat{\alpha }} \circ {\widehat{\beta }} ,\,{\widehat{\beta }} \circ {\widehat{\alpha ^{-1}}}$ and ${\widehat{\alpha ^{-1}}} \circ {\widehat{\beta ^{-1}}}$ are all embedded. Hence, the only way that ${\widehat{\mu }}$ can fail to be embedded is if one of the first two is closed. $\square$

We will now proceed to study the topology of surfaces with the generalised simple lift property.

## The topology of embedded surfaces with the generalised simple lift property

**Fig. 1:**
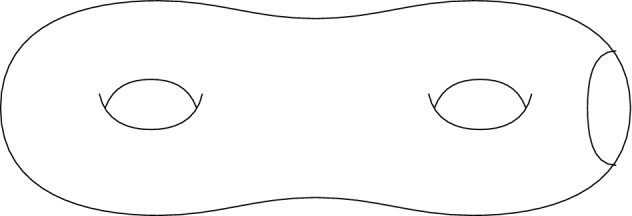
$${\mathbb {T}}^2\#{\mathbb {T}}^2\setminus {D}$$

The geometrical example at the centre of this initial topological study is the double torus minus a disk, that is the connected sum of two tori with a disk removed (see Fig. 1).

By the classification of compact surfaces, we know that compact orientable surfaces are either the sphere ${\mathbb {S}}^2$ or the connected sum of *n* tori, ${\mathbb {T}}^2\#\cdots \#{\mathbb {T}}^2$, while non-orientable surfaces are given by the connected sum if *n* projective planes ${\mathbb {R}}P^2\#\cdots \#{\mathbb {R}}P^2$. This classification extends to non-compact surfaces by taking into consideration boundary components.

### Remark 4.1

In order to simplify the notation, we will denote by ${\mathbb {T}}^2_n$ the connected sum of *n* tori, and by ${\mathbb {R}}P^2_n$ the connected sum of *n* projective planes.

### Lemma 4.2

Let $\Sigma$ be an embedded surface with the generalised simple lift property. Then two smooth, non-separating Jordan curves $\gamma _1$ and $\gamma _2$ intersecting transversally at exactly one point in $\Sigma$ cannot both have a closed $\delta$- lift into any $\Delta =\Delta (\gamma _1,\gamma _2)$, for any $\delta >0$.

### Proof

Let $\gamma _1,\,\gamma _2:[0,1]\rightarrow \Sigma$ be two smooth non-separating Jordan curves intersecting transversally at a single point *p*, that is $p=\gamma _1(0)=\gamma _1(1)=\gamma _2(0)=\gamma _2(1)$.

Arguing by contradiction, let us assume that both curves admit closed $\delta$-lifts on an embedded disk $\Delta (\gamma _1,\gamma _2)$. In other words, there exists a choice of $\delta >0$ and $U\subset \Sigma$ pre-compact open subset containing both $\gamma _1$ and $\gamma _2$, such that the embedded disk $\Delta =\Delta (\gamma _1,\gamma _2)$ given by Definition 3.1 contains ${\widehat{\gamma _1}}$ and ${\widehat{\gamma _2}}$ two closed $\delta$-lifts of $\gamma _1$ and $\gamma _2$ respectively.

Let us then consider the generalised simple lift of ${\gamma _1}\cup {\gamma _2}$ on $\Delta (\gamma _1,\gamma _2)$ pointed at (*p*, *q*). We have therefore constructed two simple closed curves ${\widehat{\gamma _1}}$ and ${\widehat{\gamma _2}}$ contained in an embedded disk $\Delta =\Delta (\gamma _1,\gamma _2)$ that intersect transversally in a single point $q\in \Delta (\gamma _1,\gamma _2)$.

This represents a contradiction to the mod 2 degree theorem applied to the Jordan-Brouwer separation theorem. This contradiction finishes the proof of the lemma.

$$\square $$

**Fig. 2:**
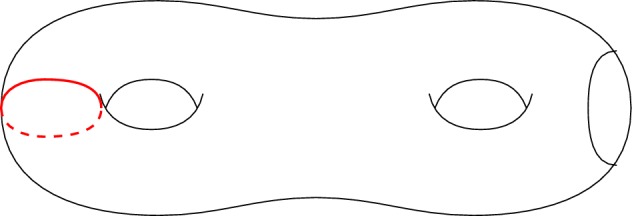
$$\gamma _1$$

**Fig. 3:**
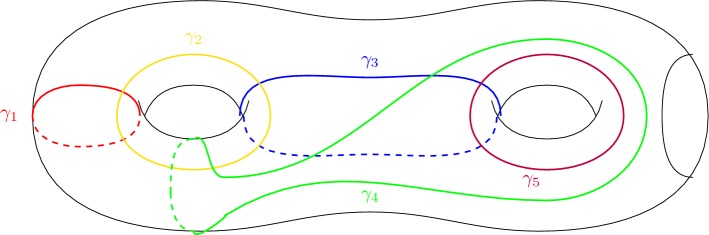
The five loops taken into consideration

In the following claims, the surface $\Sigma \subset \Omega$ that we are considering is homeomorphic to ${\mathbb {T}}^2\#{\mathbb {T}}^2\setminus {D}$ and $\gamma _1:[0,1]\rightarrow \Sigma$ denotes the smooth, non-separating Jordan curve in Fig. 2 . We will prove that a surface with the generalised simple lift property cannot contain an open subset homeomorphic to a double torus minus a disk by proving that $\gamma _1$ cannot have either a closed or an open lift in an embedded disk $\Delta$ for a specific choice of five non-separating Jordan curves $\gamma _1,\gamma _2,\gamma _3,\gamma _4,\gamma _5$ (see Fig. 3).

### Claim 4.3

Let $\Sigma \subset \Omega$ be an embedded surface homeomorphic to ${\mathbb {T}}^2\#{\mathbb {T}}^2\setminus D$ with the generalised simple lift property, and let us take into consideration the five smooth, non-separating Jordan curves $\gamma _1,\gamma _2,\gamma _3,\gamma _4,\gamma _5:[0,1]\rightarrow \Sigma$ given in Fig. 3. Then $\gamma _1$ does not admit a closed $\delta$-lift on the embedded disk $\Delta =\Delta (\gamma _1,\gamma _2,\gamma _3,\gamma _4,\gamma _5)$ given by Definition 3.1, for any $\delta >0$ and for any $U\subset \Sigma$ pre-compact open set that contains the curves.

### Proof

Given the five smooth Jordan curves $\gamma _1,\gamma _2,\gamma _3,\gamma _4,\gamma _5:[0,1]\rightarrow \Sigma$ pictured in Fig. 3, for an arbitrary $\delta >0$ and for an arbitrary pre-compact open set $U\subset \Sigma$ that contains these five curves $\gamma _i$, we are considering the embedded disk $\Delta =\Delta (\gamma _1,\gamma _2,\gamma _3,\gamma _4,\gamma _5)$ for which the connected component of $\Delta \cap {\mathcal {N}}_\epsilon (U)$ that contains the $\delta$-lifts ${\widehat{\gamma _i}}$ is a $\delta$-cover of *U* (see Definition 3.1).

Arguing by contradiction, let us assume that $\gamma _1$ admits a closed $\delta$-lift on such a disk $\Delta =\Delta (\gamma _1,\gamma _2,\gamma _3,\gamma _4,\gamma _5)$.

By Lemma 4.2, we already know that $\gamma _2$ cannot admit a closed $\delta$-lift on this disk, since $\gamma _1$ and $\gamma _2$ intersect transversally at a single point, and $\gamma _1$ has a closed lift on $\Delta$ by assumption.

Let us now consider the third curve $\gamma _3:[0,1]\rightarrow \Sigma$.

If $\gamma _3$ admitted an open lift on $\Delta$ then we would have two Jordan curves intersecting transversally at a single point $\lbrace p\rbrace =\gamma _2\cap \gamma _3$, and both admit an open $\delta$-lift on $\Delta =\Delta (\gamma _1,\gamma _2,\gamma _3,\gamma _4,\gamma _5)$. Moreover, we can take as a two-sided pre-compact subset that contains $\gamma _2$ and $\gamma _3$ the original subset $U\subset \Sigma$ used to construct $\Delta$. By the lifting lemma (Proposition 3.4), we know that the curve $\alpha :=\gamma _2\circ \gamma _3\circ \gamma _2^{-1}\circ \gamma _3^{-1}$ admits a closed $\delta$-lift on $\Delta =\Delta (\gamma _1,\gamma _2,\gamma _3,\gamma _4,\gamma _5)$, and that one of the curves $\alpha ,\gamma _2\circ \gamma _3$ and $\gamma _3\circ \gamma _2^{-1}$ has an embedded closed lift on $\Delta$.

If either $\gamma _2\circ \gamma _3$ or $\gamma _3\circ \gamma _2^{-1}$ has an embedded closed lift on $\Delta$, then we reach a contradiction by applying the same reasoning used in Lemma 4.2, since $\gamma _1$ (which we are assuming has a closed lift on $\Delta$) and the given curve intersect transversally in a single point: $\lbrace p\rbrace =\gamma _1\cap \gamma _2\circ \gamma _3$ or $\lbrace p\rbrace =\gamma _1\cap \gamma _3\circ \gamma _2^{-1}$.

If instead $\alpha =\gamma _2\circ \gamma _3\circ \gamma _2^{-1}\circ \gamma _3^{-1}$ admits an embedded closed lift, then one can find three values $t_1,t_2,t_3\in (0,1)$ for which $p=\gamma _1(t_1)=\gamma _2(t_2)=\gamma _2^{-1}(t_3)$. Following the construction of the lifting lemma, we consider the two-sided subset $U\subset \Sigma$ which in particular contains $\gamma _1,\gamma _2$ and $\gamma _3$, and pick a small simply-connected neighbourhood *V* of *p* contained in *U*, so that we can construct a family of parallel components of the lifts of *V* that can be ordered by height: $\Pi _\Sigma ^{-1}(V)\cap \Delta =\lbrace {\widehat{V}} (1),\ldots ,{\widehat{V}} (n)\rbrace$. We can now consider the generalised simple $\delta$-lift ${\widehat{\gamma _1}} \cup {\widehat{\alpha }}$ of $\gamma _1\cup \alpha$ pointed at $(p,{\widehat{p}} )$, where ${\widehat{p}} ={\widehat{\gamma _1(t_1)}} ={\widehat{\gamma _2(t_2)}} \in {\mathcal {N}}_\epsilon (p)\cap \Delta (\gamma _1,\gamma _2,\gamma _3,\gamma _4,\gamma _5)$.

The fact that ${\widehat{p}}$ is the only point of intersection results from the following remark. ${\widehat{\gamma _1}}$ is indeed a one-cover of $\gamma _1$, while on the other hand ${\widehat{\gamma _2^{-1}(t_3)}} \in {\widehat{\alpha }}$ belongs to a components of $\Pi _\Sigma ^{-1}(V)\cap \Delta$ that is different to that of ${\widehat{p}} ={\widehat{\gamma _1(t_1)}} ={\widehat{\gamma _2(t_2)}}$. In fact, if we denote by ${\widehat{V}} (l_1)$ the component of $\Pi _\Sigma ^{-1}(V)\cap \Delta$ that contains ${\widehat{p}}$, we have that the component that contains ${\widehat{\gamma _2^{-1}(t_3)}}$ will have height $l_2$ given by:

$$\begin{aligned} l_2=l_1+m[\gamma _3]\ne l_1 \end{aligned}$$

since ${\widehat{\gamma _3}}$ is an open lift on $\Delta (\gamma _1,\gamma _2,\gamma _3,\gamma _4,\gamma _5)$.

Therefore, we constructed two closed curves ${\widehat{\gamma _1}}$ and ${\widehat{\alpha }}$ which intersect transversally on the disk $\Delta =\Delta (\gamma _1,\gamma _2,\gamma _3,\gamma _4,\gamma _5)$ in a single point ${\widehat{p}}$, which represents a contradiction to the mod 2 degree theorem applied to the Jordan Brouwer separation theorem.

Therefore, the $\gamma _3$ must have a closed $\delta$-lift on $\Delta =\Delta (\gamma _1,\gamma _2,\gamma _3,\gamma _4,\gamma _5)$.

Let us now take into consideration the loop $\gamma _4$ which intersects $\gamma _2$ transversally in one single point. Arguing like before, we obtain that $\gamma _4$ will have a closed $\delta$-lift on $\Delta =\Delta (\gamma _1,\gamma _2,\gamma _3,\gamma _4,\gamma _5)$ too.

We have then constructed two smooth non-separating Jordan curves $\gamma _3$ and $\gamma _4$ that intersect transversally in one single point and both have a closed $\delta$-lift on the disk $\Delta$. By Lemma 4.2, this represents a contradiction to the Jordan Brouwer separation theorem.

This implies that the initial curve $\gamma _1$ cannot have a closed $\delta$-lift on the embedded disk $\Delta =\Delta (\gamma _1,\gamma _2,\gamma _3,\gamma _4,\gamma _5)$. $\square$

### Claim 4.4

Let $\Sigma \subset \Omega$ be an embedded surface homeomorphic to ${\mathbb {T}}^2\#{\mathbb {T}}^2\setminus D$ with the generalised simple lift property, and let us take into consideration the five smooth, non-separating Jordan curves $\gamma _1,\gamma _2,\gamma _3,\gamma _4,\gamma _5:[0,1]\rightarrow \Sigma$ given in Fig. 3. Then $\gamma _1$ does not admit an open $\delta$-lift on the embedded disk $\Delta =\Delta (\gamma _1,\gamma _2,\gamma _3,\gamma _4,\gamma _5)$ given by Definition 3.1, for any $\delta >0$ and for any $U\subset \Sigma$ pre-compact open set that contains the curves.

### Proof

Just like in the previous claim, we are working with the same five curves in Fig. 3, and for any arbitrary $\delta >0$ and for an arbitrary pre-compact open set $U\subset \Sigma$ that contains these curves $\gamma _i$ we are considering the embedded disk $\Delta =\Delta (\gamma _1,\gamma _2,\gamma _3,\gamma _4,\gamma _5)$ given by Definition 3.1, for which the connected component of $\Delta \cap {\mathcal {N}}_{\epsilon }(U)$ that contains the $\delta$-lifts ${\widehat{\gamma _i}}$ is a $\delta$-cover of *U*.

Arguing by contradiction like before, we will now assume that $\gamma _1$ admits an open $\delta$-lift on such a disk $\Delta =\Delta (\gamma _1,\gamma _2,\gamma _3,\gamma _4,\gamma _5)$.

Let us consider the curve $\gamma _2:[0,1]\rightarrow \Sigma$ which intersects $\gamma _1$ transversally in a single point. We will be considering the two cases of $\gamma _2$ admitting either an open or a closed $\delta$-lift on $\Delta =\Delta (\gamma _1,\gamma _2,\gamma _3,\gamma _4,\gamma _5)$, and we will prove that both of them yield a contradiction.

Let us first assume $\gamma _2$ admits a closed $\delta$-lift on $\Delta =\Delta (\gamma _1,\gamma _2,\gamma _3,\gamma _4,\gamma _5)$. By Lemma 4.2, this implies that both $\gamma _3,\gamma _4:[0,1]\rightarrow \Sigma$ will have an open $\delta$-lift on $\Delta$, since each one of them intersects transversally $\gamma _2$ in a single point.

Let us then consider $\gamma _5:[0,1]\rightarrow \Sigma$, which intersects $\gamma _3$ transversally in a single point. We will see that $\gamma _5$ cannot admit either an open or a closed $\delta$-lift on $\Delta =\Delta (\gamma _1,\gamma _2,\gamma _3,\gamma _4,\gamma _5)$.

If $\gamma _5$ admitted an open lift, the curves $\gamma _3$ and $\gamma _5$ would satisfy the hypotheses of the lifting lemma, and we could then apply the same argument as in the previous claim to the two curves $\gamma _2$ and $\gamma _3\circ \gamma _5\circ \gamma _3^{-1}\circ \gamma _5^{-1}$, both of which have a closed lift on $\Delta$ and intersect transversally at a single point, hence obtaining a contradiction.

If instead $\gamma _5$ admitted a closed lift, then the two curves $\gamma _3$ and $\gamma _4$ would satisfy the hypotheses of the lifting lemma, and we could apply still the same argument as in Claim 4.3 to the two curves $\gamma _5$ and $\gamma _3\circ \gamma _4\circ \gamma _3^{-1}\circ \gamma _4^{-1}$, both of which have a closed lift on $\Delta$ and intersect transversally at a single point, and hence obtain a contradiction.

These arguments imply that such a curve $\gamma _5$ cannot have either an open or a closed lift on $\Delta$, which is a contradiction, meaning that $\gamma _2$ cannot admit a closed lift.

Let us now study the case where this curve $\gamma _2:[0,1]\rightarrow \Sigma$ admits an open $\delta$-lift on $\Delta =\Delta (\gamma _1,\gamma _2,\gamma _3,\gamma _4,\gamma _5)$.

This implies that $\gamma _3:[0,1]\rightarrow \Sigma$ cannot have a closed lift on $\Delta$. In fact, if it did, we would be able to apply the lifting lemma to $\gamma _1$ and $\gamma _2$, and following the same reasoning as in Claim 4.3 to the curves $\gamma _3$ and $\gamma _1\circ \gamma _2\circ \gamma _1^{-1}\circ \gamma _2^{-1}$, we would obtain a contradiction.

Moreover, the fact that $\gamma _3:[0,1]\rightarrow \Sigma$ admits an open lift on $\Delta$ also implies that $\gamma _5:[0,1]\rightarrow \Sigma$ has an open lift on $\Delta$. This simply follows from the fact that we would be able to apply the lifting lemma to the two curves $\gamma _2$ and $\gamma _3$. So if $\gamma _5$ had a closed lift on $\Delta$, we would obtain two curves $\gamma _2\circ \gamma _3\circ \gamma _2^{-1}\circ \gamma ^{-1}_3$ and $\gamma _5$ intersecting transversally at one single point, both admitting a closed $\delta$-lift on $\Delta$, which is a contradiction as already shown in the previous claim.

We are therefore left to study the scenario where the four curves $\gamma _1,\gamma _2,\gamma _3$ and $\gamma _5$ all have an open lift on $\Delta =\Delta (\gamma _1,\gamma _2,\gamma _3,\gamma _4,\gamma _5)$.

By applying the lifting lemma to the pairs of curves $\lbrace \gamma _1,\,\gamma _2\rbrace$ and $\lbrace \gamma _3,\,\gamma _5\rbrace$ respectively, we obtain two curves with a closed lift on $\Delta =\Delta (\gamma _1,\gamma _2,\gamma _3,\gamma _4,\gamma _5)$, namely $\alpha :=\gamma _1\circ \gamma _2\circ \gamma _1^{-1}\circ \gamma _2^{-1}$ and $\beta :=\gamma _3\circ \gamma _5\circ \gamma _3^{-1}\circ \gamma _5^{-1}$.

As already pointed out in the previous claim, considering the first couple of curves $\lbrace \gamma _1,\gamma _2\rbrace$, one of the curves $\alpha$, $\gamma _1\circ \gamma _2$ and $\gamma _2\circ \gamma _1^{-1}$ has an embedded closed lift, and likewise for the other couple $\lbrace \gamma _3,\gamma _5\rbrace$. In this proof, we will take into consideration only the most complicated case where $\alpha$ and $\beta$ are the loops with the embedded closed lift on $\Delta$. One should argue just like in Claim 4.3 for the other cases.

Let us take into consideration the point of intersection $\lbrace p\rbrace =\alpha \cap \beta$. One should notice that there exist four values $t_1,t_2,t_3,t_4\in (0,1)$ such that

$$\begin{aligned} p=\gamma _2(t_1)=\gamma _2^{-1}(t_2)=\gamma _3(t_3)=\gamma _3^{-1}(t_4)\,. \end{aligned}$$

Let us now take as a two-sided pre-compact open set $U\subset \Sigma$ that contains both curves $\alpha$ and $\beta$, the original subset $U\subset \Sigma$ used to construct $\Delta =\Delta (\gamma _1,\gamma _2,\gamma _3,\gamma _4,\gamma _5)$. Following the construction in the lifting lemma, we can pick a small simply-connected neighbourhood $V\subset U$ of *p*, so that we can produce a family of parallel components on $\Delta$ of the lifts of *V* that can be ordered by height: $\Pi _\Sigma ^{-1}(V)\cap \Delta =\lbrace {\widehat{V}} (1),\ldots ,{\widehat{V}} (n)\rbrace$.

All the curves ${\widehat{\gamma _i}}$ are the open lifts, so that - still following the notation of the lifting lemma - $m[\gamma _j]\ne 0\;\forall \,j=1,\ldots ,4\,$, which means that there are two cases: either $m[\gamma _1]\cdot m[\gamma _5]>0$, or $m[\gamma _1]\cdot m[\gamma _5]<0$.

In the first case, we will consider the generalised simple lift ${\widehat{\alpha }} \cup {\widehat{\beta }}$ based at $(\gamma _2(t_1)=\gamma _3(t_3),{\widehat{p}} )$ on the disk $\Delta$, which means there exists at least a point ${\widehat{p}} \in {\mathcal {N}}_{\epsilon }(p)\cap \Delta$ such that ${\widehat{p}} \in {\widehat{\gamma _2}} \cap {\widehat{\gamma _3}}$.

We are left to prove that this point ${\widehat{p}}$ is the only point of intersection between ${\widehat{\alpha }}$ and ${\widehat{\beta }}$. By construction, ${\widehat{\gamma _2(t_1)}}$ and ${\widehat{\gamma _3(t_3)}}$ belong to the same component of $\Pi _\Sigma ^{-1}(V)\cap \Delta$, namely ${\widehat{V}} (l_1)$. The other two points ${\widehat{\gamma _2^{-1}(t_2)}}$ and ${\widehat{\gamma ^{-1}_3(t_4)}}$ will then belong to two other components ${\widehat{V}} (l_2)$ and ${\widehat{V}} (l_3)$ respectively. Moreover, since the number of components between ${\widehat{\alpha }} [k](0)$ and ${\widehat{\alpha }} [k](t)$ does not depend on *k*, we have that the heights of these two components will be:

$$\begin{aligned} l_2&=l_1-m[\gamma _1]\,,\\ l_3&=l_1+m[\gamma _5]\,. \end{aligned}$$

Hence $l_3-l_2=m[\gamma _1]+m[\gamma _5]\ne 0\,$, since we assumed $m[\gamma _1]\cdot m[\gamma _5]>0$, which means that ${\widehat{p}}$ is indeed the only point of intersection between the two closed lifts ${\widehat{\alpha }}$ and ${\widehat{\beta }}$, which is a contradiction.

In the second case, where $m[\gamma _1]\cdot m[\gamma _5]<0$, we can repeat the same argument as before, applying it to the generalised simple lift of ${\widehat{\alpha }} \cup {\widehat{\beta }}$ based at $((\gamma _2)(t_1)=\gamma _3^{-1}(t_4),{\widehat{p}} )$ instead. By using the same notation as the first case, ${\widehat{\gamma _2(t_1)}}$ and ${\widehat{\gamma _3^{-1}(t_4)}}$ will belong to the same component of $\Pi _\Sigma ^{-1}(V)\cap \Delta$, ${\widehat{V}} (l_1)$. The other two points ${\widehat{\gamma _2^{-1}(t_2)}}$ and ${\widehat{\gamma _3(t_3)}}$ will then belong to the components ${\widehat{V}} (l_2)$ and ${\widehat{V}} (l_3)$ respectively. Therefore, the heights of these two components will be given by:

$$\begin{aligned} l_2&=l_1-m[\gamma _1]\,,\\ l_3&=l_1-m[\gamma _5]\,. \end{aligned}$$

Therefore $l_3-l_2=m[\gamma _1]-m[\gamma _5]\ne 0\,$, since we assumed $m[\gamma _1]\cdot m[\gamma _5]<0$, which means that ${\widehat{p}}$ is indeed the only point of intersection between the closed lifts ${\widehat{\alpha }}$ and ${\widehat{\beta }}$, so we obtain another contradiction.

Hence we have proved that the curve $\gamma _2:[0,1]\rightarrow \Sigma$ cannot have either an open or a closed lift on the disk $\Delta$, which is a contradiction, implying that $\gamma _1$ cannot admit an open $\delta$-lift on $\Delta =\Delta (\gamma _1,\gamma _2,\gamma _3,\gamma _4,\gamma _5)$. $\square$

From these claims, we obtain the following result.

### Proposition 4.5

Given an embedded surface $\Sigma \subset \Omega$ with the generalised simple lift property, $\Sigma$ cannot contain an open subset that is homeomorphic ${\mathbb {T}}^2\#{\mathbb {T}}^2\setminus {D}$.

### Proof

The result follows directly from Claims 4.3 and 4.4. $\square$

By the classification of compact surfaces, we have that orientable surfaces are homeomorphic to ${\mathbb {S}}^2$ or the connected sum of *n* tori, ${\mathbb {T}}^2_n$, while non-orientable compact surfaces are homeomorphic to the connected sum of *n* projective planes, ${\mathbb {R}}P^2_n$. Moreover, one should notice that in the non-orientable case we have the following homeomorphisms:

- ${\mathbb {R}}P^2_2\cong K$ where *K* is the Klein bottle, and
- $${\mathbb {R}}P^2_3\cong {\mathbb {T}}^2\#{\mathbb {R}}P^2\cong K\#{\mathbb {R}}P^2$$;

which means that ${\mathbb {R}}P^2_{2k}\cong {\mathbb {T}}^2_{k-1}\#K$ and ${\mathbb {R}}P^2_{2k+1}\cong {\mathbb {T}}^2_k\#{\mathbb {R}}P^2$.

Proposition 4.5 then gives the following result.

### Corollary 4.6

If $\Sigma \subset \Omega$ is an embedded compact surface and has the generalised simple lift property, then it must be topologically ${\mathbb {S}}^2$, ${\mathbb {T}}^2$, ${\mathbb {R}}P^2$, ${\mathbb {R}}P^2_2\cong K$, ${\mathbb {R}}P^2_3\cong {\mathbb {T}}^2\#{\mathbb {R}}P^2$ or ${\mathbb {R}}P^2_4\cong {\mathbb {T}}^2\#K$.

## Minimal laminations

Let us now apply the results of the previous section to the case of minimal laminations. Let us first recall some facts about laminations.

### Definition 5.1

A subset ${\mathcal {L}}\subset \Omega$ is a *smooth lamination* if for each $p\in {\mathcal {L}}$, there is a radius $r_p>0$, maps $\phi _p,\psi _p:{\mathcal {B}}_{r_p}(p)\rightarrow {\mathbb {B}}_1(0)\subset {\mathbb {R}}^3$ and a closed set $T_p\subset (-1,1)$ with $0\in T_p$ such that:

1. $$\phi _p(p)=\psi (p)=\mathbf {0}$$;
2. $\phi _p$ is a smooth diffeomorphism and ${\mathbb {D}}_1(0)\subset \phi _p\big ({\mathcal {L}}\cap {\mathcal {B}}_{r_p}(p)\big )$;
3. $\psi _p$ is a Lipschitz diffeomorphism and ${\mathbb {B}}_1(0)\cap \lbrace x_3=t\rbrace _{t\in T_p}=\psi _p({\mathcal {L}}\cap {\mathcal {B}}_{r_p}(p))$;
4. $$\phi _p^{-1}({\mathbb {D}}_1(0))=\psi ^{-1}_p({\mathbb {D}}_1(0))\,.$$

We refer to maps $\phi _p$ satisfying properties 1) and 2) as *smoothing maps* of ${\mathcal {L}}$ and to maps $\psi _p$ satisfying properties 1) and 3) as *straightening maps* of ${\mathcal {L}}$.

A smooth lamination ${\mathcal {L}}\subset \Omega$ is *proper* in $\Omega$ if it is closed, that is $\overline{{\mathcal {L}}}={\mathcal {L}}$. Any embedded smooth surface is a smooth lamination that is proper if and only if the surface is proper.

### Definition 5.2

Let ${\mathcal {L}}\subset \Omega$ be a non-empty smooth lamination. A subset $L\subset {\mathcal {L}}$ is a *leaf of *${\mathcal {L}}$ if *L* is a connected, embedded surface and for any $p\in L\,,\exists \, r_p>0$ and a smoothing map $\phi _p$ so that ${\mathbb {D}}_1=\phi _p(L\cap {\mathcal {B}}_{r_p}(p))\,.$ For each $p\in {\mathcal {L}}$, we will denote by $L_p$ the unique leaf of ${\mathcal {L}}$ containing *p*.

A smooth lamination ${\mathcal {L}}$ is a *minimal lamination* if each one of its leaves is minimal.

The following is the natural compactness result for sequences of properly embedded minimal surfaces with uniformly bounded second fundamental form (see for instance Appendix B in [6] for a proof).

### Theorem 5.3

Let $\lbrace \Sigma _i\rbrace _{i\in {\mathbb {N}}}$ be a sequence of smooth minimal surfaces, properly embedded in $\Omega$. If for each compact subset $U\subset \Omega$ there is a constant $C(U)<\infty$ so that

$$\begin{aligned} \sup _{U\cap \Sigma _i}\vert A_{\Sigma _i}\vert \le C(U)\,, \end{aligned}$$

then, $\forall \,\alpha \in (0,1)$, up to passing to a subsequence, the $\Sigma _i$s converge in $C^{\infty ,\alpha }_{loc}(\Omega )$ to ${\mathcal {L}}$, a smooth proper minimal lamination in $\Omega$.

### Remark 5.4

While the straightening maps converge in $C^\alpha$, their Lipschitz norms are uniformly bounded on compact subsets of $\Omega$. This follows from the Harnack inequality and is used in the proof of Theorem 5.3 (see Appendix B of [6] and Theorem 1.1 in [16]).

In view of the result in Theorem 5.3, one can define the so-called *singular points* of a sequence ${\mathcal {S}}:=\lbrace \Sigma _i\rbrace _{i\in {\mathbb {N}}}$ of properly embedded smooth minimal surfaces $\Sigma _i$.

### Definition 5.5

Given the sequence ${\mathcal {S}}=\lbrace \Sigma _i\rbrace$, we define the *regular* points to be the set of points

$$\begin{aligned} \mathrm {reg}({\mathcal {S}}):=\bigg \lbrace p\in \Omega \mid \exists \,\rho >0\text { such that } \limsup _{i\rightarrow \infty }\sup _{B_\rho (p)\cap \Sigma _i}\vert A_{\Sigma _i}\vert <\infty \bigg \rbrace \end{aligned}$$

and the *singular* points of ${\mathcal {S}}$ to be the set

$$\begin{aligned} \mathrm {sing}({\mathcal {S}}):=\bigg \lbrace p\in \Omega \mid \forall \,\rho >0\text { such that } \limsup _{i\rightarrow \infty }\sup _{B_\rho (p)\cap \Sigma _i}\vert A_{\Sigma _i}\vert =\infty \bigg \rbrace \,. \end{aligned}$$

Clearly, $\mathrm {reg}({\mathcal {S}})$ is an open subset of $\Omega$, while $\mathrm {sing}({\mathcal {S}})$ is closed in $\Omega$. In general, $\mathrm {sing}({\mathcal {S}})\subset \Omega {\setminus }\mathrm {reg}({\mathcal {S}})$ is a strict inclusion, however, by Lemma I.1.4 in [6] there exists a subsequence ${\mathcal {S}}'$ of ${\mathcal {S}}$ so that $\Omega =\mathrm {reg}({\mathcal {S}}')\cup \mathrm {sing}({\mathcal {S}}')$. Without loss of generality, we will then consider sequences ${\mathcal {S}}$ that admit this decomposition.

This work will be centred around limit laminations of minimal disk sequences, so it will be convenient to introduce the following definition (inspired by [17]).

### Definition 5.6

Let us take a closed set $K\subset \Omega$ in our ambient Riemannian three-manifold $\Omega$. Let us introduce a smooth proper minimal lamination ${\mathcal {L}}$ in $\Omega \setminus K$ and a sequence ${\mathcal {S}}=\lbrace \Sigma _i\rbrace _{i\in {\mathbb {N}}}$ of properly embedded minimal disks in $\Omega$.

We will refer to the quadruple $(\Omega ,K,{\mathcal {L}},{\mathcal {S}})$ as a *minimal disk sequence* if

i. $$\mathrm {sing}({\mathcal {S}})=K$$, and
ii. $\Sigma _i\setminus K$ converge to ${\mathcal {L}}$ in $C^{\infty ,\alpha }_{loc}(\Omega \setminus K)$, for some $\alpha \in (0,1)$.

The case where the $\Sigma _i$ are assumed to be disks has been extensively studied and some structural results have been proved on the possible singular sets *K* and limit laminations ${\mathcal {L}}$ of a minimal disk sequence $(\Omega ,K,{\mathcal {L}},{\mathcal {S}})$. For example, in [3–6] Colding and Minicozzi show that *K* must be contained in a Lipschitz curve and that for any point $p\in K$ there exists a leaf of ${\mathcal {L}}$ that extends smoothly across *p*.

When $\Omega ={\mathbb {R}}^3$, they further show that either $K=\emptyset$ or ${\mathcal {L}}$ is a foliation of ${\mathbb {R}}^3\setminus K$ by parallel planes and that *K* consists of a connected Lipschitz curve which meets the leaves of ${\mathcal {L}}$ transversely. Using this result, Meeks and Rosenberg showed in [14] that the helicoid is the unique non-flat properly embedded minimal disk in ${\mathbb {R}}^3$. This uniqueness was then used by Meeks in [13] to prove that if $\Omega ={\mathbb {R}}^3$ and $K\ne \emptyset$, then *K* is a line orthogonal to the leaves of ${\mathcal {L}}$, which is precisely the limit of a sequence of rescalings of a helicoid.

For an arbitrary Riemannian three-manifold, such a simple description is not possible. In [2], Colding and Minicozzi construct a sequence of properly embedded minimal disks in the unit ball ${\mathbb {B}}_1(0)\subset {\mathbb {R}}^3$ which has $K=\lbrace 0\rbrace$ and whose limit lamination consists of three leaves: two non-proper disks that spiral into the third, which is the punctured unit disk in the $x_3$-plane. Inspired by this example, more cases have been constructed where the singular set *K* consists of any closed subset of a line [7, 9, 11, 12], as well as examples where *K* is curved [15]. Finally, Hoffman and White [10] have also constructed minimal disk sequences in which $K=\emptyset$ and the limit lamination ${\mathcal {L}}$ has a leaf which is a proper annulus in $\Omega$.

### Proposition 5.7

Leaves of a minimal disk sequence in $\Omega$ have the generalised simple lift property.

### Proof

Given *L* a leaf of ${\mathcal {L}}$, if *L* is a disk, the curves $\gamma _i$ in *L* are themselves their own simple $\delta$-lifts in any pre-compact open set $U\subset L$ that contains them. Hence the proposition holds trivially, with $q=p$.

In the more general case, when *L* is not a disk, it is sufficient to prove the existence of a generalised simple lift of a single curve $\gamma$. By Proposition B.1 in Appendix B of [6], we obtain a bound on the Lipschitz norms of the straightening maps, which implies that for each pre-compact open subset $U\subset L$, there is a constant $C=C(U)$ such that $C\lambda \in (0,1)$, and then for each $\Sigma _i\in {\mathcal {S}}$, ${\mathcal {N}}_\lambda (U)\cap \Sigma _i$ is a (possibly empty) $C\lambda$-graph over *U*. Given a curve $\gamma :[0,1]\rightarrow L$ contained in an open pre-compact subset $U\subset L$, let us denote by *l* the length of $\gamma$ and *d* the diameter of *U*. For any $\delta >0$, choose $\epsilon >0$ such that $C\epsilon <\min \lbrace 1,\delta \rbrace$. Let $\mu =\frac{3}{4}\exp (-2C(l+d))$ and pick $\Sigma _\mu \in {\mathcal {S}}$ such that ${\mathcal {N}}_{\mu \epsilon }(p)\cap \Sigma _\mu \ne \emptyset$, where $p=\gamma (0)$. Let $\Gamma$ be a component of $\Sigma _\mu \cap {\mathcal {N}}_\epsilon (U)$ which contains a point $q\in {\mathcal {N}}_{\mu \epsilon }(p)\cap \Gamma$. We have chosen $\epsilon >0$ so that $\Sigma _\mu \cap {\mathcal {N}}_{\epsilon }(U)$ is a $\delta$-graph over *U*. We claim that $\Gamma$ is a $\delta$-cover of *U* containing a $\delta$-lift of $\gamma$. This follows by showing that any curve in *U* of length at most $2(l+d)$ starting at *p* has a lift in $\Gamma$ starting at *q*. By construction, this lift is necessarily a $\delta$-lift.

Indeed, if $\sigma :[0,T]\rightarrow U$ is parametrised by arclength, and ${\widehat{\sigma }} :[0,T']\rightarrow \Gamma$ satisfies $\Pi _L({\widehat{\sigma }} (t))=\sigma (t)$ for some $0<T'<T$, then

$$\begin{aligned} \bigg \vert \frac{d}{dt} dist^{\Omega }\big (\sigma (t),{\widehat{\sigma }} (t)\big )\bigg \vert \le C\,dist^{\Omega }\big (\sigma (t),{\widehat{\sigma }} (t)\big ) \end{aligned}$$

and so

$$\begin{aligned} dist^{\Omega }\big (\sigma (t),{\widehat{\sigma }} (t)\big )\le \exp (Ct)\cdot dist^{\Omega }(p,q)<\epsilon \mu \,\exp (Ct)<\epsilon \,, \end{aligned}$$

where the last inequality follows from the fact that $t\le T\le l+d$. Furthermore, if $t<T$, then the lift ${\widehat{\sigma }} (t)$ may be extended past *t* provided $dist^\Omega \big (\sigma (t),{\widehat{\sigma }} (t)\big )<\epsilon$, which proves that leaves of a minimal disk sequence have the generalised simple lift property as claimed. $\square$

This result then implies:

### Proposition 5.8

If *L* is an embedded compact surface obtained as a leaf of a minimal disk sequence $(\Omega ,K,{\mathcal {S}},{\mathcal {L}})$ then it must be topologically ${\mathbb {S}}^2$, ${\mathbb {T}}^2$, ${\mathbb {R}}P^2$, ${\mathbb {R}}P^2_2\cong K$, ${\mathbb {R}}P^2_3\cong {\mathbb {T}}^2\#{\mathbb {R}}P^2$ or ${\mathbb {R}}P^2_4\cong {\mathbb {T}}^2\#K$.

### Remark 5.9

By applying a lifting argument, one can further rule out the sphere ${\mathbb {S}}^2$ and the projective plane ${\mathbb {R}}P^2$.

Combining together Proposition 5.8 and the previous remark, we then obtain the following result.

### Corollary 5.10

An embedded compact surface *L* obtained as a leaf of a minimal disk sequence $(\Omega ,K,{\mathcal {S}},{\mathcal {L}})$ must be topologically ${\mathbb {T}}^2$, ${\mathbb {R}}P^2\cong K$, ${\mathbb {R}}P^2_3\cong {\mathbb {T}}^2\#{\mathbb {R}}P^2$ or ${\mathbb {R}}P^2_4\cong {\mathbb {T}}^2\#K$.
